# Pilot study for the evaluation and adaptation of a Four Item-Acne-Scar Risk Assessment Tool (4-ASRAT): a resource to estimate the risk of acne-induced scars

**DOI:** 10.12688/f1000research.23737.1

**Published:** 2020-06-26

**Authors:** Jorge Estrella Porter, Mikaela Camacho, María Isabel Viteri, Katherine Aguilar, Drifa Belhadi, Vincenzo Bettoli, Anita del Rocío Buestán, Brigitte Dréno, Pablo Endara, Alison Layton, Nelly Machado, Rosa Mateus, Jerry Tan, Enrique Terán, Paola Yépez, Jonathan Guillemot

**Affiliations:** 1 Escuela de Medicina, Universidad San Francisco de Quito USFQ, Quito, Pichincha, 170901, Ecuador; 2 Health Research Group, Universidad San Francisco de Quito USFQ, Quito, Pichincha, 170901, Ecuador; 3 SISTEMAS MÉDICOS SIME, Universidad San Francisco de Quito USFQ, Quito, Pichincha, 170157, Ecuador; 4 Hôpitaux Universitaires Paris Nord Val de Seine (Bichât Claude Bernard), Paris, 75018, France; 5 Azienda Ospedaliero-Universitaria di Ferrara, Ferrara, 44122, Italy; 6 Centre Hospitalier Universitaire de Nantes, Nantes, 44000, France; 7 Harrogate and District NHS Foundation Trust, Harrogate, HG2 7SX, UK; 8 University of Western Ontario, London, N6A 5B9, Canada

**Keywords:** acne, acne scars, prevention tool, risk factors, scarring, scarring prevention

## Abstract

**Background:** Acne-induced scarring is associated with a similar burden as acne, i.e. diminished quality of life, and may be avoided if patients receive appropriate and timely acne treatment. In 2017, a four item-Acne-Scar Risk Assessment Tool (4-ASRAT) was designed by Tan *et al*. to categorise patients with acne into lower-risk or higher-risk for acne scarring. Its applicability outside the initial study population (France, Brazil and United States) remains to be determined.

**Methods:** A study protocol was developed to create a systematic approach for validating and adapting 4-ASRAT to different populations, Ecuador in this case. The protocol was reviewed by 11 local and international dermatologists and pilot-tested in an Ecuadorian population using a sample of 10 participants who currently had or had had acne. Feedback from the pilot study was used to improve the study protocol. The results of the pilot study are included here, and the final study protocol is available as extended data.

**Results:** The protocol proved to be applicable. Images taken of participants were a valuable resource for dermatological evaluation about the presence or absence of acne scars. Tangential light is necessary for this evaluation. Although dermatological assessments varied, we concluded that assessment by three local dermatologists for each participant was adequate for reaching a consensus on the presence or absence of acne scars.

**Conclusions:** Considering the morbidity related to acne and acne scars, tools designed as prevention that alert patients about risk of developing scarring are necessary. The proposed protocol shows a feasible way of validating and adapting 4-ASRAT to different populations.

## Introduction

Acne is among the most common dermatological conditions, with an estimated prevalence among the general population of 9.4% ^1^. Prevalence among those aged 12 to 24 years can reach as high as 85%, with variations in different populations ^2^. While acne can be perceived as a trivial condition due to its temporary nature, the emotional and psychological burden is high ^3^. Patients with acne have diminished quality of life ^4^, and are more likely to suffer from depression and anxiety ^5^. The extent of these impacts are comparable to that of patients with chronic disability associated with asthma, epilepsy, diabetes or arthritis ^6, 7^.

Acne often leads to the development of scars, which can be permanent ^8^. Acne scarring is also associated with a psychosocial burden similar to that seen with acne ^9^. Scarring may, however, be avoided if patients receive appropriate and timely acne treatment ^10– 12^. Although risk factors for the development of acne scars are well-known, only one tool exists to predict the risk of acne scarring to support treatment initiation decision-making ^13^.

In 2017, Tan *et al.* ^13^ described the first, and to our knowledge only, tool which assesses the risk for acne scarring in patients with acne based on four major risk factors: “severity of acne”, “family history of acne scarring”, “squeezing and picking behaviours” and “duration of acne”. For simplicity, we named this tool the Four-item Acne Scar Risk Assessment Tool (4-ASRAT). The tool is a short questionnaire, which can be either self-administered or administered by a healthcare worker. Using a score associated with each item to the questionnaire and a score threshold, 4-ASRAT provides a binary outcome on the risk of acne scarring by categorising respondents as being at “lower” or “higher risk”. 4-ASRAT was calibrated and validated using cross-validation based on a pre-existing database containing a large sample of young adults from the United States, France and Brazil, and resulted in a sensitivity of 82% and specificity of 43%. However, its applicability to other populations is uncertain.

This study presents a protocol for validating and, if necessary, adapting 4-ASRAT to any population. The proposed protocol was tested via a pilot study in an Ecuadorian population to test its applicability and obtain feedback to develop a final version. With this study, we intend to develop and disseminate a protocol for the adaptation of 4-ASRAT to other populations, thereby promoting best practices for timely acne care and acne scar prevention.

## Methods

This study consisted of two phases: first, the development of a study protocol for the evaluation and adaptation of 4-ASRAT to different populations and, second, the pilot of the said protocol in a real-world scenario in Quito, Ecuador, to obtain feedback and improve the proposed protocol. A study using the final protocol in a large Ecuadorean population will be the subject of a subsequent publication.

### Protocol development

The protocol aimed to provide a replicable process to evaluate and adapt 4-ASRAT to any population. It was conceived as a reviewed version of the initial study protocol proposed by Tan *et al*. To be effective, the protocol must provide a strategy to answer three questions: What set of risk factors should the adapted 4-ASRAT use? What score should be associated with each item? What score threshold should it use?

In addition to the nature of the information that it must lead to, the study protocol must address cost-effectiveness. The adaptation of 4-ASRAT must remain simple for it to be put in practice: the application of the protocol should be inexpensive, in terms of time, human resources and money. This means that the study protocol should also be readily available and provide adequate detail to ensure replicability.

As part of the protocol, a 22-question survey regarding acne scaring risk factors, epidemiology and self-perception of acne scars was developed (available as *Extended data*). Based on acne scar prevalence, the sample of respondent should be at least 250 participants ^14^. This questionnaire, which was not formally assessed in this pilot study, is designed to establish the significant risk factors related to acne scars for the evaluation and possibly the adaptation of 4-ASRAT. Participants should go to a photobooth immediately after filling the questionnaire (characteristics of the photobooth details available as *Extended data*) ^15^, where three pictures will be taken for each participant (front, and right and left profiles). A group of independent dermatologists then evaluate participants’ pictures to determine the presence or absence of acne scars. Their evaluation will be considered as the gold standard for data analysis.

Data will be computed and calibration and discrimination of the tool will be calculated to validate 4-ASRAT in the studied population. If 4-ASRAT proves invalid for the given population, the adaptation process then begins. Based on the questionnaire results and dermatologists’ evaluations, risk factors for acne scaring and their respective weight to determine the relevant list of risk factors, the scores associated with each response and the optimal score threshold. The complete version of the protocol can be consulted as *Extended data* ^16^.

### Piloting approach

The pilot study was conducted to evaluate the feasibility of the aforementioned study protocol and improve its design before a full-scale research conduct. Besides the study of the data collected as part of the application of the standard study protocol, additional information was collected using observation of the data collection in real-world settings and by obtaining feedback from dermatologists.

In practice, the pilot consisted of a small-scale application of the study protocol with particular attention to the data collection process and a data analysis approach benefitting from expert input.

A sample of 10 participants, which is among the range recommended for pilot studies ^17^, was recruited by an open invitation through social media and flyers in Universidad San Francisco de Quito USFQ, during October 2018, using the same inclusion criteria as in the protocol:

- To be a person aged 18–25 at the time of participation.
- To have suffered acne at any time point, including having active acne at the time of the study.
- To belong to [sample pool population]
- To accept signing the informed consent, including consent for photographs to be captured.
- To have no visible facial hair at the moment of the photograph
- To have no facial make-up at the moment of the photograph

Participants answered the 22-question survey and were then taken to the photobooth for the three necessary photographs. Data collected as part of the pilot was recorded and tabulated as planned in the study protocol. Participants photographs were showed to a total of 11 dermatological professionals (seven local dermatologists and four international experts) for the evaluation of the presence or absence of acne scars in participants. This process was conceived to determine the minimum number of dermatologists needed to reach a consensus in the evaluation of the presence or absence of acne scars. The following inclusion criteria were used for dermatologists:

- To be a medical doctor with a specialty degree in dermatology.
- To have at least five years of experience in seeing patients with acne.
- To have provided medical attention to at least 35 acne patients per year over the last 5 years.
- To consent to the project participation an accept the workload proposed.
- Preferred: to have at least one publication related to acne in a scientific journal.

Dermatologists provided feedback on photography quality to improve the final study protocol. Besides elementary descriptive statistics, no statistical analysis was necessary at the stage of the pilot to evaluate the applicability of the protocol.

### Ethical considerations

The study protocol and pilot study were approved by Universidad San Francisco de Quito’s Institutional Review Board (Comité de Ética de Investigación en Seres Humanos Universidad San Francisco de Quito) on September 25 ^th^ 2018 (2018-193IN). Informed consent was obtained from each participant.

## Results

### Piloting results

The pilot study was carried out in October 2018 with a sample of ten participants recruited from students of Universidad San Francisco de Quito USFQ (six women and four men, aged 18–25, mean age: 23 years). Results about the evaluation of the presence or absence of acne scars are shown in Table 1.

**Table 1.** Summary of the dermatological evaluation findings in the pilot study.

Participant code	Participant Evaluation	Local dermatologist evaluation							Expert evaluation			
		D1	D2	D3	D4	D5	D6	D7	D8	D9	D10	D11
01-P	P	*p*	P	P	A	A	A	A	A	A	A	A
02-P	P	P	*p*	P	*p*	*p*	*p*	*p*	*p*	*p*	*p*	*p*
03-P	A	A	A	A	A	P	A	A	A	A	A	A
04-P	P	*p*	P	P	*p*	*p*	*p*	A	*p*	*p*	*p*	*p*
05-P	A	*p*	A	P	A	*p*	*p*	A	*p*	A	*p*	*p*
06-P	A	A	A	*p*	A	A	A	A	A	*p*	A	A
07-P	A	*p*	A	*p*	P	A	*p*	*p*	*p*	P	*p*	*p*
08-P	A	P	*p*	P	*p*	*p*	P	*p*	P	*p*	P	*p*
09-P	A	A	P	P	A	A	*p*	A	*p*	*p*	A	A
10-P	P	P	*p*	P	P	P	*p*	*p*	*p*	*p*	*p*	*p*

D: dermatologist; P: acne scars are present, more than mild; *p*: Scars are present, but mild; A: acne scars are absent. This table presents the results of the protocol pilot with ten participants evaluated by 11 dermatologists, of whom seven were local dermatologists and four were international acne expert dermatologists (expert in table). For participant 01-P, the participant’s self-evaluation is that acne scars are present. Local dermatologist D1 evaluated that mild acne scares are present, local dermatologists D2 and D3 reported that acne scars beyond mild are present and local and expert dermatologists D4 to D11 reported that acne scars are absent. A simple majority showed to be equally effective in professional evaluation than more complex systems of determining consensus with compound majorities.

Participants and dermatologists were able to complete the study without issues. Scarring assessments varied widely: compared to the gold standard established by international experts – who generally agreed – local dermatologists had a little more difficulty agreeing, with consensus reached for 6 out of 10 cases vs. 9 out of 10 for international experts. When a consensus was reached among local dermatologists, the outcome always concurred with that of international experts. This suggests that local dermatologists are an adequate proxy for international experts provided a minimum number of participating local dermatologists, which we established at three.

Participant self-evaluations varied more widely from the expert gold standard: half the time self- and international expert assessments concurred. When they differed, participants were more likely to minimize the presence of scars (false negatives, 3/10) rather than exaggerate (false positives, 1/10).

Dermatologists generally validated the approach for photographing, with three main recommendations reached by consensus:

- Tangential light is mandatory to assess scar volume and depth in photography.
- Besides from front, left and right angle photographs, an oblique picture of the participants should be included.
- Make-up, even if invisible, should not be allowed in participants.

Surveys and photographs were easily collected.

### Final protocol

Similar to Tan *et al*.’s study, the protocol proposes a methodology for studying at a single timepoint younger adults (18–25 years), and considers their history of acne and acne scarring in order to identify acne-scarring risk factors and their respective weight. Depending on the context, incentives may be used to promote participation. The study is designed to be completed in a six-month timeframe. All of the recommendations given by dermatologists were considered for its final version, and three dermatologists were established as the minimum number to reach consensus. No further adaptations to the protocol were required based on the pilot study.

## Discussion

The proposed protocol showed applicability and ease of execution, while confirming its usefulness in obtaining the necessary data for the validation and adaptation of 4-ASRAT (epidemiologic data, self-evaluation about presence or absence of acne scars, associated risk factors, and images for dermatological evaluation). However, some limitations were found.

Although preferable for accuracy, a prospective study including younger participants (children who have not developed acne scars yet) with follow-up evaluations was avoided for practical reasons: a study with these characteristics is impractical because of the time it implies in terms of follow-up, resulting in a low probability of applying the protocol in other populations.

A single-site study was also chosen (a university) to take advantage of the concentration of eligible volunteers who were willing to participate. The study centre should be selected, if possible, based on the representativeness of the sample pool compared to the general population and should include individuals not only from a single institution (university) but rather more representative of the population’s context.

Recruiting a representative population sample is a complex and costly exercise, which seems unrealistic in the context of acne-scar prevention. Although the lack of representativeness of the sample proposed in the study protocol may be perceived as a limitation, we prefer the local and cost-effective adaptation of 4-ASRAT to the use of the non-calibrated tool. Researchers should seek aggregated groups, such as universities, which are the best proxies to the general population for the adaption study.

Participant photography will never equal the quality of face-to-face evaluation, as bidimensional imaging does not yet allow volumetric assessment, a necessary element for determining the severity of a suspected scar ^18^. Tangential light must be included in the final study, to obtain a more three-dimensional image of participants, as suggested by dermatologists during the pilot study. Again, we prefer the more cost-effective approach using photography, despite its limitations, rather than professional individual evaluations, which increase the study costs significantly.

Regarding scar assessments, comparing self- to local professional assessments, the pilot suggests the need for dermatological assessments rather than reliance on self-assessment. As opposed to an earlier study, the pilot results hint towards a tendency of participants to minimise the presence of acne scars rather than overestimate, which was earlier found ^19^. It was shown that a simple majority consensus is sufficient to determine the presence or absence of acne scars, so the use of three dermatologists is recommended for final evaluation (minimum number needed to reach a simple majority consensus).

## Conclusions

We developed and piloted a readily available study protocol to evaluate and adapt 4-ASRAT to any population. We showed this protocol to be applicable in practice, provided that certain precautions were taken, including photography quality and local dermatologist support. Due to the scarcity of tools to assess the risk of acne scaring, the use of an adapted and validated tool for prediction of acne scar risk in a particular population is a valuable public health measure.

## Data availability

### Underlying data

All data underlying the results are available as part of the article and no additional source data are required.

### Extended data

Harvard Dataverse: Questionnaire for the validation and adaptation of a tool to estimate the risk of acne-induced scars in different population. https://doi.org/10.7910/DVN/WGDWB0 ^15^.

This project contains the questionnaire to be given to participants.

Harvard Dataverse: Protocol for the validation and adaptation of a tool to estimate the risk of acne-induced scars in different populations. https://doi.org/10.7910/DVN/NED0GS ^16^.

This project contains the protocol to be conducted for the main study.

Extended data are available under the terms of the Creative Commons Zero "No rights reserved" data waiver (CC0 1.0 Public domain dedication).
