# Supplementary material for: Pilot study for the evaluation and adaptation of a Four Item-Acne-Scar Risk Assessment Tool (4-ASRAT): a resource to estimate the risk of acne-induced scars
Source: F1000Res. 2020 Jun 26;9:651. [Version 1] doi: 10.12688/f1000research.23737.1 (PMC7431969; doi:10.12688/f1000research.23737.1)
Supplement: Peer review history

# Peer review: Reviewer response for version 1

**DOI:** 10.5256/f1000research.26191.r68895

**Published:** 2020-08-17

**Authors:** Zhou Bing-Rong

**Affiliations:** 1 Department of Dermatology, The First Affiliated Hospital of Nanjing Medical University, Nanjing, China

I think 4-ASRAT will be a useful and powerful tool for assessing whether acne scars occur in patients with acne lesions. As we all know, the treatment of these scars is still full of challenges. Therefore, if there is a simple and easy way to assess the risk of scar formation in acne patients and take early prevention measures, it may help to reduce the incidence of acne scars.

 The current 4-ASRAT questionnaire is relatively suitable for rapid assessment of acne scar formation risk in a short period of time. Although it has good predictive sensitivity and specificity, I think the questionnaire still needs to add more specific indicators. It is better to be able to evaluate under the guidance of dermatologists. If the assessment items can include risk tools to predict the formation of acne atrophic scar, hypertrophic scar and keloid respectively, it will help doctors to deal with the corresponding problems. In addition, because in this study, according to the patient's facial lesions photos, in some cases, both dermatology experts and local dermatologists did not reach a high degree of consistency, which indicates that the definition of acne scar is still vague. Although it is very difficult to reach 100% consensus, it is important to make a more appropriate definition of acne scar before the assessment and to train the assessors for a certain period of time.

Is the work clearly and accurately presented and does it cite the current literature?

Yes

If applicable, is the statistical analysis and its interpretation appropriate?

Yes

Are all the source data underlying the results available to ensure full reproducibility?

Yes

Is the study design appropriate and is the work technically sound?

Yes

Are the conclusions drawn adequately supported by the results?

Yes

Are sufficient details of methods and analysis provided to allow replication by others?

Yes

Reviewer Expertise:

Phototherapy.

I confirm that I have read this submission and believe that I have an appropriate level of expertise to confirm that it is of an acceptable scientific standard.

# Peer review: Reviewer response for version 1

**DOI:** 10.5256/f1000research.26191.r65623

**Published:** 2020-08-03

**Authors:** Vicente Torres Lozada, Maria Fernanda Torres-Calderón

**Affiliations:** 1 Instituto Tecnológico y de Estudios Superiores de Monterrey, Mexico City, Mexico; 2 Dermatology Department, Hospital Juarez, Mexico City, Mexico

Regarding the 4 ASRAT tool, I believe it can be good for screening the risk of developing scars, as tools with good sensitivity and moderate specificity are. It could be helpful as early intervention to prevent the detriment of their self esteem and mental health. The extended questionnaire, if applied to patients who already have acne, it could be helpful to intervene as soon as possible, under the premise that they are prone to develop acne scars or if they already have a few, so they do not develop any more scarring. This tool could translate into better adherence to treatment and less scarring, as well as knowing they are prone to develop them, for their future offspring. 

 Regarding patients who already have acne scars, I think this tool will only show that if left untreated they will develop more scars.

 I believe the 4 ASRAT tool could incorporate questions from the extended questionnaire, but those specifically regarding risk factors for developing inflammatory acne, and the questionnaire should be applied by either a physician or a healthcare personnel, in order to standardize and reassure the questions are adequately interpreted by the patient. This could perhaps be done at the primary care physician office, with a referral to dermatology if the patient has a high risk of developing scars. Also, the full questionnaire would take around 20 minutes, so perhaps the most relevant questions should be kept to make it a 5-7 minute questionnaire or if most questions should remain the questionnaire should be made by trained personnel to make it objective and quicker.

Is the work clearly and accurately presented and does it cite the current literature?

Yes

If applicable, is the statistical analysis and its interpretation appropriate?

Yes

Are all the source data underlying the results available to ensure full reproducibility?

Yes

Is the study design appropriate and is the work technically sound?

Yes

Are the conclusions drawn adequately supported by the results?

Yes

Are sufficient details of methods and analysis provided to allow replication by others?

Yes

Reviewer Expertise:

Acne and Rosacea, Acne scars.

We confirm that we have read this submission and believe that we have an appropriate level of expertise to confirm that it is of an acceptable scientific standard.
